# Long-term follow-up after treatment of tubercular uveitis: case series and review of the literature

**DOI:** 10.3389/fopht.2023.1270948

**Published:** 2023-11-17

**Authors:** Ikhwanuliman Putera, Paul L. A. van Daele, Josianne C. E. M. ten Berge, Willem A. Dik, Rina La Distia Nora, P. Martin van Hagen, Saskia M. Rombach

**Affiliations:** ^1^ Department of Ophthalmology, Erasmus University Medical Center, Rotterdam, Netherlands; ^2^ Department of Internal Medicine, Section Allergy and Clinical Immunology, Erasmus University Medical Center, Rotterdam, Netherlands; ^3^ Laboratory Medical Immunology, Department of Immunology, Erasmus University Medical Center, Rotterdam, Netherlands; ^4^ Department of Ophthalmology, Faculty of Medicine, Universitas Indonesia – Cipto Mangunkusumo Hospital, Jakarta, Indonesia; ^5^ Department of Internal Medicine and Immunology, Rotterdam Eye Hospital, Rotterdam, Netherlands

**Keywords:** anti-tubercular treatment, immunosuppressive, recurrence, tubercular uveitis, follow-up

## Abstract

**Introduction:**

There is a scarcity of long-term follow-up data and management strategies for recurrent uveitis in tubercular uveitis (TBU), especially in cases extending beyond 10 years after the completion of initial antitubercular treatment (ATT).

**Methods:**

This retrospective study involved five TBU patients who were initially treated with a combination of four-drug ATT for 6 months, and the five of them had more than 10 years of follow-up after uveitis resolution upon ATT completion. We describe the occurrence of recurrent uveitis and present our approach to managing these recurrent episodes.

**Results:**

Recurrent uveitis and cystoid macular edema (CME) developed in three out of five included TBU patients with a median of 18 years (range 13–20 years) of follow-up. The anatomical sites of the recurrences were anterior, intermediate, and pan-uveitis. The recurrent episodes varied from 6 years to 15 years after ATT completion. Systemic or local corticosteroids/immunosuppressants successfully resolved all recurrent episodes, but one was also treated with the combination of isoniazid monotherapy again. Two patients needed anti-tumor necrosis factor-α therapy.

**Conclusion:**

Long-term monitoring of TBU patients after ATT completion is warranted. Further well-designed studies with larger sample sizes are required to better estimate the risk of recurrences, investigate the underlying mechanism of recurrences, and identify biomarkers that predict who is at risk for recurrences.

## 1 Introduction

Diagnosis, treatment, and the preferred time for follow-up after initial anti-tubercular treatment (ATT) remain challenges in tubercular uveitis (TBU). The term TBU, as defined by a group of experts named the Collaborative Ocular Tuberculosis Study (COTS), includes intraocular inflammation associated with active or latent *Mycobacterium tuberculosis* (*Mtb*) infection (1). However, prescribing ATT for uveitis patients with a positive interferon-gamma release assay (IGRA) and/or tuberculin skin test (TST) without proven pulmonary or other extrapulmonary TB is often problematic (2). Even if a patient fits the definition mentioned above, not all uveitis spectra under the umbrella term of TBU have achieved consensus to initiate anti-tubercular treatment (ATT) (2). When there is doubt about the diagnosis, Gupta et al. have proposed criteria that included a response to ATT as part of the clinical clues toward an appropriate diagnosis of TBU (3). A recent head-to-head meta-analysis showed that, compared with those treated with corticosteroid only, TBU patients who received ATT had fewer recurrences (4). International guidelines focus on diagnostic criteria and the initial treatment; however, the long-term prognosis of TBU patients is not yet taken into account. The COTS group defined “cure” as “inactive disease (grade 0 cells/no inflammation) 24 months after a complete course of ATT.” The inability to taper systemic corticosteroids to < 10 mg/day or topical steroid drops to < 2 drops/day and the continuation of steroid-sparing immunosuppressants that are sometimes necessary after the initial treatment do not fit into these criteria (1). To date, reports on the long-term outcome of TBU beyond 24 months after treatment are scarce. We herein present our long-term follow-up observation of TBU patients treated with ATT. We describe the occurrence of recurrent uveitis and its management.

## 2 Methods

This is a retrospective study based on medical records data of TBU patients treated and monitored at the Department of Ophthalmology, Erasmus Medical Center, and the Rotterdam Eye Hospital, Rotterdam, Netherlands, between January 2002 and January 2004. Specifically, this study provides a follow-up report of more than 10 years on TBU patients who received ATT and were previously included in a TBU patient cohort (5). The diagnosis was based on active uveitis with a positive Mantoux/TST and no other cause of uveitis based on clinical presentation and ancillary tests (5). IGRA testing was not yet available at that time. All patients completed 6 months of ATT, consisting of 2 months of isoniazid, rifampicin, pyrazinamide, and ethambutol, followed by 4 months of isoniazid, rifampicin, and ethambutol (2HRZE/4HRE). None of the reported cases tested positive for human immunodeficiency. Neither side effects nor non-compliance with ATT were noted in any of the patients.

For this study, recurrence was defined as an increment of intraocular inflammation that required treatment adjustment. Cystoid macular edema (CME) was identified with optical coherence tomography (OCT) macula images showing any intraretinal/subretinal fluid.

## 3 Results

In the previous case series (5), seven TBU patients were treated with ATT. However, two of them lacked follow-up: one patient had a positive culture result for TB on a lymph node biopsy but passed away 1 year following ATT due to a complication of a severe pemphigoid unrelated to TB or TB treatment; the other patient underwent vitrectomy due to retinal detachment 2 years after ATT but did not have any follow-up visits. The remaining five patients had long-term follow-up with a median follow-up of 18 years (range 13–20 years). Of the remaining five patients with long-term follow-up (**Table 1**), recurrence of uveitis occurred in three of them, and these specific cases are described in more detail below.

**Table 1:** Demographic profile, clinical presentation at initial treatment, and follow-up condition of included patients.

Case	Age category at ATT start (in years)	Place of birth and category of TB incidence*	Laterality of initial inflammation	Initial inflammation (anatomical site)	Duration of follow-up	Recurrence during follow-up	Anatomical site, in case of recurrent inflammation	CME during follow-up	Medications received during follow-up for recurrence/CME	Condition at last follow-up
**1**	40–45	Outside the Netherlands, lower-moderate	Bilateral	Posterior (unspecific choroidal lesion with vitritis)	20 years	Yes, at 8 years and 11 years after ATT as bilateral uveitis	Panuveitis	Yes	First recurrent episode *Initial treatment: Isoniazid 1 × 300 mg/day and systemic steroid (start prednisone at 50 mg/day, then taper gradually) for 4 months *Subsequent treatment: Triamcinolone acetonide subconjunctival injection (nine times for the right eye and five times for the left eye) Second recurrent episode Infliximab infusion (for 1 year), and mycophenolate mofetil (until the last visit; 10 years in total) Last visit treatment Topical dexamethasone three times a day for the left eye and mycophenolate mofetil 2 × 500 mg Other medication and its indication: hypertension (under control with clonidine 0.025 mg)	No inflammation/CME in either eyes BCVA of the right eye: 0.02 (corneal decompensation due to viral keratitis in the follow-up) BCVA of the left eye: 0.2
**2**	45–50	Outside the Netherlands, lower-moderate	Unilateral	Posterior (non-occlusive retinal vasculitis with CME)	13 years	No	Not applicable	No	No anti-inflammatory drugs/drops Other medication: topical antiglaucoma (dorzolamide/timolol) for open-angle glaucoma, unrelated to TBU	No inflammation/CME in either eye BCVA of the right eye: 1.00 BCVA of the left eye: 1.00
**3**	35–40	Outside the Netherlands, upper-moderate	Bilateral	Anterior (chronic non-granulomatous)	19 years	No	Not applicable	No	No anti-inflammatory drugs/drops Other medication: topical lubricant as needed for dry eyes	No inflammation/CME in either eyes BCVA of the right eye: 1.00 BCVA of the left eye: 0.80 (cataract)
**4**	50–55	Outside the Netherlands, lower-moderate	Bilateral	Posterior (serpiginous-like choroiditis)	18 years	Yes, at 8 years (right eye) and 15 years (both eyes) after ATT	Anterior	Yes	First and second recurrent episodes Subconjunctival betamethasone injections (total of two injections) and topical prednisolone acetate 1% (up to six times daily) Last visit treatment Topical prednisolone acetate 1% once a day in both eyes Other medication and its indication: none	No inflammation/CME in either eye BCVA of the right eye: 0.7 BCVA of the left eye: 1.0
**5**	18–20	Netherlands, low	Bilateral	Intermediate (dense vitritis with snowballs and CME)	14 years	Yes (bilateral) at 6 years after ATT	Intermediate	Yes	Recurrent episode Mycophenolate mofetil and then combined with adalimumab for 5 years Last visit treatment Topical lubricant as needed Other medication and its indication: none	No inflammation/CME in either eye BCVA of the right eye: 0.5 BCVA of the left eye: 1.2

ATT, anti-tubercular treatment; BCVA, best corrected visual acuity; CME, cystoid macular edema; TBU, tubercular uveitis.

*Categorization based on the incidence per 100,000 population per year in 2019 [available from: https://cdn.who.int/media/docs/default-source/hq-tuberculosis/who_globalhbcliststb_2021-2025_backgrounddocument.pdf?sfvrsn=f6b854c2_9 (accessed on: 28 August 2023)].

### Case 1

A patient presented with bilateral posterior uveitis and was free of inflammation for 8 years after ATT. The recurrent uveitis at 8 years’ follow-up appeared as a more severe bilateral intraocular inflammation (panuveitis with edematous optic nerve) than the initial inflammation. In the time interval between ATT completion and the recurrent episode, the patient had two travel visits to a country with lower-moderate TB incidence category (**Table 1**) but had no history of close contact with a TB case. At the time of recurrence, the systemic workup only revealed a high QuantiFERON^®^-TB Gold In-tube level (15.09) without any other signs of active systemic TB. The patient received 300 mg of isoniazid daily and oral prednisolone (starting at 50 mg daily with gradual tapering) for 4 months, and clinical resolution was achieved. The next recurrent episode presented 11 years after the initial presentation. No other cause of infectious or non-infectious uveitis was noted at the time of recurrent uveitis, and the patient was successfully treated with mycophenolate mofetil and infliximab. Further details of the treatment strategies and duration for the management of recurrent uveitis episodes are provided in **Table 1**. Until the end of the follow-up, 20 years after the initial presentation, the patient still used mycophenolate mofetil and topical dexamethasone three times a day for the left eye.

### Case 2

A patient with unilateral non-occlusive retinal vasculitis with CME did not show a relapse of uveitis or recurrent CME after ATT during the follow-up period of 13 years (**Table 1**).

### Case 3

This case, presenting with chronic bilateral non-granulomatous anterior uveitis, did not show a relapse of uveitis or CME after ATT during the follow-up period of 19 years (**Table 1**).

### Case 4

A case presented with bilateral serpiginous-like choroiditis. After completing ATT and corticosteroid treatment, the inflammation had completely subsided. Recurrent episodes of anterior uveitis occurred after 8 years and 15 years of follow-up. Repeated systemic laboratory workup showed no abnormalities that could be contributing to the recurrent uveitis, except for the high QuantiFERON-TB Gold In-tube level (9.89). Based on the discretion of the ophthalmologist and immunologist at that time, the patient was then treated with only subconjunctival steroid injections (betamethasone, twice) and topical 1% prednisolone acetate (**Table 1**). No inflammation was noted at the last follow-up visit, 18 years after the initial presentation.

### Case 5

This particular case presented with bilateral intermediate uveitis and CME. The uveitis was completely resolved after ATT. The recurrent disease episode presented 6 years after ATT as bilateral intermediate uveitis. No other infectious or non-infectious causes of uveitis were noted after repeated systemic workup at the time of recurrent uveitis. An IGRA test was not performed at that time. The patient was treated with mycophenolate mofetil, after which adalimumab was started due to unresolved ocular inflammation under the former drug. The treatment for recurrent uveitis took 5 years until complete resolution of inflammation was achieved (**Table 1**). From that time to the last visit at 14 years’ follow-up, there was neither recurrence nor CME with complete discontinuation of systemic and local anti-inflammatory drugs.

(Place **Table 1**)

## 4 Discussion

Recurrent uveitis can even appear many years following the completion of ATT. Three out of five patients developed recurrence, of whom two needed a systemic immunosuppressant and a biologic. From the available literature, the reported follow-up duration following ATT treatment in TBU was limited. Only some studies reported the treatment outcome, including recurrent uveitis, after 2 years of observation (**Table 2**). Unlike for new uveitis cases, there is no standardized guideline for the workup of recurrences. Laboratory investigations could help to rule out other differential diagnoses, such as autoimmune-related uveitis (11). Even though testing of ocular fluid for *Mtb* detection is important for establishing the diagnosis of TBU, especially in countries where TB is not endemic, the diagnosis mainly relies on clinical presentation, the exclusion of other potential entities, and a positive IGRA or TST result (12). In current case series, in none of the cases was another alternative systemic disease or infection identified that might have contributed to the development of recurrent episodes of uveitis. With the broader applicability of the IGRA test nowadays, IGRA is applied more often, compared with the TST (13). In a previous study, conducted in a non-endemic TB setting, the level of the IGRA was not associated with specific clinical phenotypes in TB uveitis (14). Moreover, the outcome of ATT treatment in TB uveitis was not predicted by the level of the IGRA at initial presentation (14). The level of IGRA does not seem to be an adequate biomarker for latent TB treatment (15). Taken together, the decision to restart ATT might not be solely based on the level of IGRA at the time of recurrences. In case 1, tailored isoniazid monotherapy was prescribed to prevent systemic TB activation during systemic corticosteroid treatment. Although it can be difficult to exclude a reinfection with TB even after a thorough examination, in none of the cases was there a suspicion of reinfection based on the clinical history and absence of other organ involvement. Importantly, there is no specific consensus or guideline for re-initiating ATT for recurrences. Multani et al. reported that, of 15 patients with a recurrence of tubercular uveitis, two restarted ATT without additional immunosuppressant use and achieved clinical resolution (16). Meanwhile, the remaining 13 patients were treated with local/systemic corticosteroid without restarting ATT, resulting in inactive disease in five patients. The remaining eight patients required an additional immunosuppressant, of whom six patients achieved clinical resolution (16). Another study by Kawali et al. demonstrated that, in possible tubercular vasculitis patients who received ATT, 16 out of 24 experienced recurrences during a median time of follow-up of 40 months (range 3–159 months) (17). However, specific details of the treatment for recurrent episodes were not provided (17). In the case of ATT-treated tubercular serpiginous-like choroiditis, treatment with ATT significantly reduces the likelihood of recurrences, although complete elimination of recurrences is not guaranteed (8). As described, anterior uveitis can also manifest as recurrent uveitis in tubercular serpiginous-like choroiditis following complete treatment with ATT (8).

**Table 2:** Data from studies on treatment outcome in patients with tubercular uveitis patients with 2 or more years of follow-up.

No	Study	Country	Number of patients with ≥ 2 years of follow-up	Inclusion criteria of the study	Characteristics of patients	Initial treatment of TB uveitis	Duration of follow-up	Reported cases with recurrent uveitis during follow-up	Treatment of recurrent uveitis and/or macular edema during follow-up
**1**	Lee et al. (2022) (6)	Republic of Korea	One out of five	Inclusion criteria: presence of choroidal tuberculoma supporting evidence of TB infection	One patient with concomitant miliary, lymphadenitis, and meningitis TB	One patient with 2-year ATT (not described in detail) and systemic dexamethasone	7 years	None	None
**2**	Collaborative Ocular Tuberculosis Study (COTS)-1 (2020) (7)	Multiple countries	228	Inclusion criteria: clinical appearance of active uveitis exclusion of other uveitis entities positive investigation, one or more of the following: direct acid-fast bacilli smear, PCR test, TST/IGRA, or radiological test 24 months’ follow-up	O Bilateral uveitis: 152 out of 228 patients (66.7%) o Anatomical location of initial uveitis episode: * anterior uveitis (41 patients, 18.0%) * intermediate uveitis (27 patients, 11.8%) * posterior uveitis (81 patients, 35.5%) * panuveitis (76 patients, 33.3%)	ATT ± local/systemic steroid (not specified, depending on the participating center) O 143 patients (62.7%) received ATT + oral steroid O 31 patients (13.6%) did not receive ATT or steroids O 30 patients (13.2%) received ATT + oral steroid + IMT O 24 patients (10.5%) received only ATT	2 years	Not reported	Not reported
**3**	Kawali et al. (2020) (8)	India	Seven out of 18 (ATT-treated)	Patients who presented with serpiginous-like choroiditis lesions	Bilateral: two cases Unilateral: five cases	9-month ATT (± local and/or systemic IMT) * Four patients treated with methotrexate and/or azathioprine in combination with ATT	Range 28–60 months	Two patients	Not reported
**4**	Cordero-Coma et al. (2013) (9)	Spain	12 out of 17	Inclusion criteria: 1. intermediate uveitis, posterior uveitis, and panuveitis tuberculosis 2. completion of ATT 3. one of the following criteria: * active uveitis despite immunosuppressive treatment * positive IGRA or TST * exclusion of other uveitis entities after workup	Three retinal vasculitis Nine posterior uveitis (with one serpiginous-like choroiditis and two choroiditis) Two patients had active pulmonary TB	Combination of 9-month ATT and oral prednisone of maximum of 0.5 mg/kg/day (for the initial 3 months of treatment)	Range 2–5 years	Three patients	All recurrent cases were treated with IMT (two received adalimumab). The result of recurrent uveitis treatment was not reported
**5**	Docummun et al. (2012) (10)	Switzerland	12	Inclusion criteria: 1. active uveitis (granulomatous uveitis, vitritis, or choroiditis) with positive TST 2. adult patients 3. exclusion of other entities after workup	10 multifocal choroiditis and two serpiginous-like choroiditis	* Six patients received 6-month isoniazid monotherapy * One patient received three-drug combination consisting of 2RHZ/4RH * Four patients received four-drug combination consisting of 2RHZE/4RH * Two patients received no ATT Note: systemic corticosteroid in selected cases (not clearly defined)	Range 2–7 years	Two patients	Not described

ATT, anti-tubercular therapy; E, ethambutol; H, isoniazid; IGRA, interferon-gamma release assay; IMT, immunosuppressive treatment; PCR, polymerase chain reaction; R, rifampicin; TST, tuberculin skin test; Z, pyrazinamide.

Previously, three hypothetical explanations for recurrences and chronic inflammation in TBU have been coined (1): the recurrence itself is caused by non-TB etiologies that are inadequately managed with anti-inflammatory treatment (16) (2), non-viable *Mtb* (after complete ATT) could still cause intraocular inflammation (18), and (3) autoimmune response could follow *Mtb* infection (19). As hypothesized in a recent review (19), plausible autoimmunity in TB could also have occurred in the cases presented here.

To date, there is still much unknown about the pathomechanism underlying recurrent uveitis in TBU. It was shown by an animal study that the presence of dead *Mtb* in the eye could stimulate ocular inflammation presenting as panuveitis (18). Furthermore, the primed mice, which received prior intramuscular injection of dead-*Mtb*, were more likely to have a chronic course of uveitis than the unprimed ones, with a significantly higher level of IL-17, VEGF, CXCL9, CXCL10, IL-12p40, and macrophage inflammatory protein-1α (MIP-1α/CCL3) in the vitreous samples (18). Thus, the presence of an *Mtb* antigen that might persist after ATT could induce chronic ocular inflammation. However, whether this phenomenon is associated with an excessive immune response to TB-antigens, induction of an anti-retinal immune response, or both, still needs to be addressed.

Aside from the possibility of an *Mtb* antigen being present in the eye, there is also a plausible development of autoimmunity following *Mtb* infection (19). Autoimmunity following TB infection likely involves autoreactive T-lymphocytes and the formation of anti-retinal autoantibodies (ARAs). Analysis of intraocular samples from TBU patients showed the presence of anti-retinal autoreactive T-lymphocytes (20). Moreover, increased serum positivity for ARA has been described in TBU, and intraocular fluid from IGRA-positive uveitis patients more frequently contained anti-tyrosinase autoantibody that was significantly associated with CME occurrence (21, 22). However, serum ARAs, immunoglobulin G (IgG) against whole human retina extract (WHRE) and interphotoreceptor retinoid-binding protein (IRBP) were also detected at a higher level in cases of ocular toxoplasmosis and other non-infectious, immune-mediated uveitis (23). Even though serum anti-WHRE and anti-IRBP IgG levels were not as high as in those with toxoplasma and other non-infectious immune-mediated uveitis, slightly higher levels of those IgGs were also detected in individuals with chronic toxoplasma infection but without uveitis (23). The exact role of serum ARAs and the ocular inflammatory status of the eye remain poorly understood and warrant further exploration, particularly whether ARAs against specific epitopes is induced in TBU and could help differentiate TBU from other types of infectious uveitis. Furthermore, a case report was published of a bladder cancer patient who developed anterior granulomatous uveitis following intravesical bacillus Calmette–Guérin (BCG) treatment (24). Excessive cell death might play a part in autoimmunity development following *Mtb* infection (19). This inefficient, excessive cell death in microbial-infected host cells enables the presentation of self-antigens that would lead to the generation of autoreactive T-helper 17 cells (25). Interestingly, the death-receptor signaling pathway was augmented in *Mtb*-infected retinal pigment epithelium (26).

When autoimmunity plays a significant role, as comprehensively discussed in a recent review (27), long-term immunosuppressive therapy may be indicated in selected patients. Anti-TNF-α was effective in treating recurrences in this case series without any sign of systemic TB disease reactivation. However, our findings must be interpreted with caution. This is a selection of patients with long-term follow-up. We aimed to include all seven patients identified between January 2002 and January 2004; however, only five of them had long-term follow-up data available. While our initial reporting did not set specific time restrictions for follow-up, it is worth noting that all five patients were followed up for more than 10 years and reports of recurrent uveitis in such extended follow-up periods following primary treatment with ATT for TBU are scarce. It is currently unknown whether recurrence in TBU is different between high- and low-TB-burden settings. In the absence of a gold standard for diagnosing TBU (28), it is also plausible that recurrences may be inherent to the uveitis course in uveitis of an unknown cause with positive TB immunoreactivity, which may not necessarily signify true TBU caused by direct infection in the ocular tissues. In all cases, uveitis resolution was initially achieved by subsequent administration of ATT after previous inadequate response to corticosteroids, reflecting the high probability of true TBU in the presented cases. Currently, there is no adequate evidence as to whether it is best to restart ATT in the first place for treating recurrent uveitis in a high-TB-burden setting. Although treatment with ATT for more than 6 months in the management of TBU appears to result in improved outcomes (4), there is still insufficient evidence to support the initiation of ATT for a duration exceeding 6 months. Consideration of ATT with a duration of more than of 6 months may be advisable in cases with signs of active TB other than in the eyes, particularly when the central nervous system is affected, or in situations of severe initial presentation (29). Additionally, it remains unclear whether 9-month ATT would effectively reduce the risk of recurrences, which warrants future investigation. Meanwhile, most studies reported the use of 6-month ATT for the mainstay treatment of TBU (4). According to the recent consensus, maintaining inactive ocular inflammation for 2 years following ATT is considered sufficient (1); however, our observations suggest that it may be prudent not to entirely disengage from the patient. Despite our efforts to document relevant medical comorbidities during recurrent uveitis episodes, including potential metabolic conditions such as hypertension, we were unable to obtain data on smoking. While the association of smoking and other metabolic diseases with the occurrence of non-infectious anterior uveitis has been noted (30–32), a more robust investigation of their stronger connection with uveitis activity, including recurrence, needs to be addressed in larger studies. Furthermore, we did not have samples to measure cellular or autoantibody responses in these patients, as these could be of interest in elucidating mechanisms, including plausible autoinflammation or autoimmunity, and markers for recurrent episodes of uveitis in TBU.

## 5 Conclusion

This retrospective study, despite its limited sample size, raises the importance of the long-term monitoring of TBU patients and highlights the possibility of recurrences unrelated to viable *Mtb.* Recurrence of inflammation and CME may occur years after TBU has been treated with ATT, which might be difficult to treat and may necessitate long-term immunosuppressive therapy. Further well-designed studies with larger sample sizes are required to better estimate the risk of recurrences, investigate the underlying mechanism of recurrences, and identify biomarkers that predict who is at risk for recurrences.

## Data availability statement

The original contributions presented in the study are included in the article/supplementary material. Further inquiries can be directed to the corresponding author.

## Ethics statement

The studies involving humans were approved by Erasmus University Medical Center (MEC-2022-0645). The studies were conducted in accordance with the local legislation and institutional requirements. Written informed consent for participation was not required from the participants or the participants’ legal guardians/next of kin in accordance with the national legislation and institutional requirements.

## Author contributions

IP: conceptualization, data curation, formal analysis, investigation, methodology, writing—original draft, and writing—review and editing. PD: data curation, investigation, and writing—review and editing. JB: investigation, supervision, and writing—review and editing. WD: supervision, validation, and writing—review and editing. RN: supervision, validation, and writing—review and editing. PH: conceptualization, methodology, supervision, validation, and writing—review and editing. SR: conceptualization, supervision, validation, methodology, writing—review and editing, and writing—original draft.
